# Response Surface Optimization of Cellulose-Rich Material Recovery from *Carludovica palmata* Fibers: Effects on Structural and Thermal Properties

**DOI:** 10.3390/polym18182300

**Published:** 2026-09-20

**Authors:** Rogelio Antonio Canul Piste, Emilio Pérez Pacheco, Carlos Rolando Ríos Soberanis, Mario Adrián de Atocha Dzul Cervantes, Jorge Carlos Canto Pinto, Alejandro Ortiz Fernández

**Affiliations:** 1 Tecnológico Nacional de México (TecNM), Campus ITS_Calkiní (ITESCAM), Cuerpo Académico Bioprocesos, Av. Ah-Canul, Calkiní C.P. 24900, Campeche, Mexico; 10459@itescam.edu.mx (R.A.C.P.); eperez@itescam.edu.mx (E.P.P.); maadzul@itescam.edu.mx (M.A.d.A.D.C.);; 2 Centro de Investigación Científica de Yucatán, A.C (CICY), Unidad de Materiales, Calle 43, No. 130 x 32 y 34, Colonia Chuburná de Hidalgo, Mérida C.P. 97205, Yucatán, Mexico; rolando@cicy.mx

**Keywords:** *Carludovica palmata*, cellulose-rich material recovery, lignocellulosic fibers, response surface methodology, central composite design, crystallinity, thermal stability

## Abstract

This study optimized the chemical treatment conditions for *Carludovica palmata* fibers using response surface methodology (RSM) based on central composite designs (CCDs). Acid and alkaline treatments were evaluated as independent experimental routes to determine the effects of reagent concentration, temperature, and treatment time on gravimetric recovery. The resulting models identified operating conditions associated with maximum predicted recovery within each experimental domain. Maximum experimental gravimetric recoveries of 42.7% and 57.7% were obtained for the acid and alkaline treatments, respectively. Fourier transform infrared spectroscopy (FTIR), X-ray diffraction (XRD), and thermogravimetric analysis (TGA) were used to evaluate structural and thermal changes in the resulting materials. FTIR spectra showed reductions in bands associated with hemicellulose and lignin-related functionalities after chemical treatment. The Segal crystallinity index increased from 41.2% in untreated fibers to 52.7% and 63.8% in the materials obtained under the selected acid and alkaline treatment conditions, respectively. Thermal analysis further showed an increase in the maximum degradation temperature following chemical treatment. Collectively, the results demonstrate that the treatment conditions influenced both gravimetric recovery and the structural and thermal characteristics of the recovered materials. These findings support further investigation of *C. palmata* as a non-conventional lignocellulosic feedstock for cellulose-based polymer materials.

## 1. Introduction

The growing environmental burden of persistent petroleum-derived plastics has intensified interest in renewable feedstocks for developing polymeric materials [1,2]. Among the available bio-based resources, cellulose is particularly relevant because of its abundance, biodegradability, mechanical performance, and chemical versatility [3,4]. Structurally, cellulose consists of linear β-(1 → 4)-linked glucan chains organized into crystalline and amorphous domains, whose relative organization influences its physicochemical and mechanical behavior [5]. These characteristics have enabled their use in polymer composites, biodegradable films, micro and nanocellulosic materials, aerogels, and biomedical matrices.

Non-conventional lignocellulosic biomass, including tropical leaf fibers and agricultural residues, offers an alternative cellulose source that may complement established feedstocks [1,5,6]. Cellulose contents of approximately 35–65 wt.% have been reported for different lignocellulosic fibers, although their composition varies substantially with botanical origin, maturity, and processing conditions [3,7,8]. Isolating cellulose from these matrices requires disrupting the lignocellulosic network, in which cellulose is associated with hemicellulose and lignin through a complex structural arrangement that restricts its accessibility [8,9,10].

Chemical fractionation using acidic, alkaline, solvent-based, or oxidative treatments has therefore been widely investigated to isolate cellulose. Acid treatment promotes the hydrolysis and solubilization of susceptible polysaccharide fractions, whereas alkaline treatment facilitates lignin removal by disrupting ester and ether linkages within the lignin-carbohydrate network. Researchers have reported the effectiveness of these treatments for lignocellulosic sources such as rice straw, sugarcane bagasse, pea pod waste, and coconut fibers [11,12,13,14]. Nevertheless, extraction performance depends strongly on biomass composition and process severity; excessive reagent concentration, temperature, or treatment time may decrease material recovery through degradation or solubilization of biomass constituents.

Despite their effectiveness for lignocellulosic fractionation, conventional acid and alkaline treatments have environmental and operational limitations associated with corrosive chemicals, chemical consumption, water-intensive washing and neutralization steps, and the generation of acidic or alkaline wastewater [15,16]. Consequently, the environmental performance of cellulose extraction depends not only on the origin of the lignocellulosic feedstock but also on the severity and resource requirements of the fractionation process. Optimizing reagent concentration, temperature, and treatment time is therefore important for identifying operating conditions that favor material recovery while avoiding unnecessarily severe processing conditions.

Response surface methodology (RSM) provides a statistical framework for evaluating such multivariable systems. When combined with a central composite design (CCD), RSM estimates linear, interaction, and quadratic effects while reducing the number of experimental combinations needed to characterize the response surface [17]. This approach is particularly suitable for lignocellulosic fractionation, where the response to chemical treatment can depend simultaneously on reagent concentration, temperature, and treatment time. Rather than assuming that increasing process severity improves extraction, RSM allows for experimental identification of operating regions associated with favorable material recovery.

*Carludovica palmata*, commonly known as jipijapa palm, is a perennial plant native to Central and South America whose fibers have traditionally been used for artisanal weaving because of their flexibility and appearance [18]. Despite this established use, its potential as a lignocellulosic feedstock for cellulose-based materials remains comparatively underexplored. In particular, the response of *C. palmata* fibers to independently applied acid and alkaline treatments under systematically optimized conditions remains insufficiently established. This knowledge gap limits the assessment of how processing conditions affect both material recovery and the structural and thermal characteristics relevant to subsequent polymer-related applications.

Accordingly, this study aimed to evaluate and optimize independent acid and alkaline treatment routes for recovering cellulose-rich material from *C. palmata* fibers using RSM based on CCD. Reagent concentration, temperature, and treatment time were evaluated as process variables affecting gravimetric recovery, and the materials obtained under the selected conditions were characterized by Fourier transform infrared spectroscopy (FTIR), X-ray diffraction (XRD), and thermogravimetric analysis (TGA) to assess treatment-induced changes in chemical functionality, crystalline organization, and thermal behavior, respectively.

## 2. Materials and Methods

### 2.1. Materials

**Figure 1:**
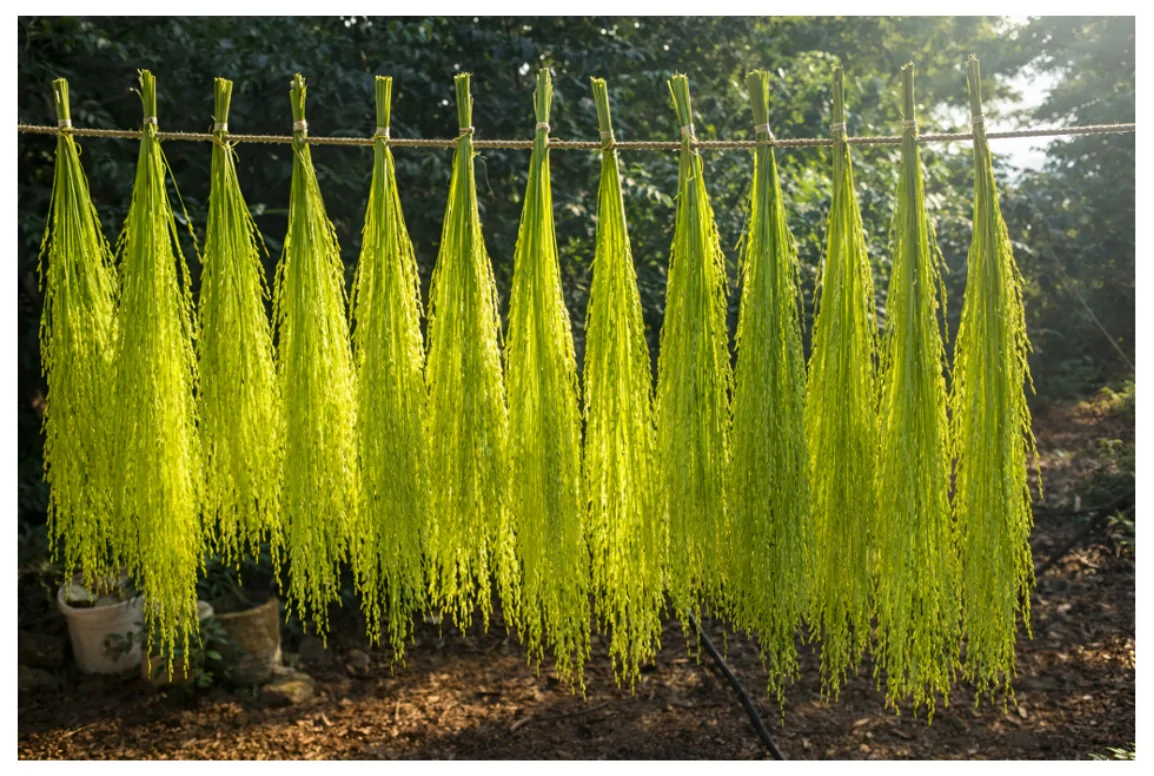
*Carludovica palmata* buds are used for fiber preparation.

Buds of *Carludovica palmata* (Figure 1) were collected between October and December 2023 in Santa Cruz, Calkiní, Campeche, Mexico (20.396292° N, 90.238138° W). A total of 100 buds were collected and transported for subsequent fiber preparation and chemical processing.

Sodium hydroxide (NaOH, 97%, Meyer, Mexico City, Mexico), sulfuric acid (H_2_SO_4_, 95–98%, Sigma-Aldrich, St. Louis, MO, USA), and sodium hypochlorite (NaClO, commercial solution, 5%) were used for the chemical treatments. All aqueous solutions were prepared using distilled water. The reagents were used as received unless otherwise specified.

### 2.2. Fiber Preparation

*C. palmata* fibers were prepared following the traditional artisanal procedure used to obtain fibers for hat weaving. The outer sheath was manually removed, then the central vein was separated. The leaf laminae were then cut into longitudinal strips and manually scraped to obtain fiber. The resulting fibers were air-dried under sunlight for four days and subsequently stored in kraft paper bags under ambient laboratory conditions until further processing.

Before chemical treatment, the fibers were manually cut into segments approximately 1 cm long, without mechanical grinding. To reduce residual moisture and standardize the initial dry mass used in the experiments, the fibers were additionally oven-dried at 60 °C for 24 h before weighing out the 35 g portions used for each experimental run.

### 2.3. Experimental Design

Response surface methodology (RSM) based on central composite designs (CCDs) was used to investigate the effects of three independent process variables on gravimetric recovery for the acid and alkaline treatment routes. The independent variables were reagent concentration (A), treatment temperature (B), and reaction time (C). Separate CCDs were established for the acid and alkaline treatments because different operating ranges were evaluated for each treatment route.

Each experimental design comprised 20 runs, including factorial, axial, and center-point runs. Table 1 and Table 2 present the actual and coded levels of the independent variables for the acid and alkaline treatments, respectively, while Table 3 and Table 4 provide the corresponding experimental matrices.

Each CCD included six replicated center-point runs, which were performed under identical combinations of reagent concentration, temperature, and treatment time. These center-point replicates were included to estimate experimental variability and pure error for gravimetric recovery. The remaining factorial and axial design points were performed as individual experimental runs according to the CCD structure.

The experimental response was the gravimetric recovery of the treated solid material, expressed as a percentage relative to the initial dry mass of pretreated fiber. The response obtained for each treatment route was fitted independently to a second-order polynomial model according to Equation (1):

**Y** = β_0_ + β_1_A + β_2_B + β_3_C + β_12_AB + β_13_AC + β_23_BC + β_11_A^2^ + β_22_B^2^ + β_33_C^2^ (1)

where **Y** is the predicted response; β_0_ is the intercept; β_1_, β_2_, and β_3_ are the linear regression coefficients; β_12_, β_13_, and β_23_ are the interaction coefficients; β_11_, β_22_, and β_33_ are the quadratic coefficients; and A, B, and C represent reagent concentration, treatment temperature, and reaction time, respectively.

### 2.4. Statistical Analysis

Statistical analyses were performed using Design-Expert^®^ software (version 7.0.0, Stat-Ease Inc., Minneapolis, MN, USA). The experimental responses obtained from the acid and alkaline treatment designs were independently fitted to second-order polynomial models. Analysis of variance (ANOVA) was used to evaluate the statistical significance of the fitted models and individual regression terms at a 95% confidence level (*p* < 0.05).

Model adequacy was evaluated using the coefficient of determination (R^2^), adjusted coefficient of determination (adjusted R^2^), and model F-value. The significance of the linear, interaction, and quadratic terms was determined from their corresponding *p*-values. Response surface and contour plots were generated from the fitted models to visualize the effects of the independent variables and their interactions on gravimetric recovery.

The replicated center points were used to estimate experimental variability (pure error). The dispersion of the center-point responses was considered when assessing model adequacy and the reliability of the fitted response surfaces.

Experimental variability in gravimetric recovery was estimated from the six replicated center points included in each CCD. These replicated runs were used to estimate pure experimental error. No additional replication beyond the CCD structure is assumed unless explicitly indicated.

Numerical optimization was performed independently for the acid and alkaline treatment models to identify the combination of reagent concentration, temperature, and reaction time associated with maximum predicted gravimetric recovery within the respective experimental domains.

### 2.5. Pretreatment

Before the CCD-defined experimental treatments, the fibers underwent alkaline pretreatment to disrupt the lignocellulosic matrix and increase access to the carbohydrate fraction. The pretreatment conditions were selected based on alkaline processing conditions previously reported for lignocellulosic fibers.

For each pretreatment batch, 35 g of dried fiber was treated with 1 L of aqueous NaOH solution (4%), corresponding to a solid-to-liquid ratio of 1:28.6 (g:mL). The suspension was stirred continuously at 1200 rpm and maintained at 100 °C for 60 min under reflux. After treatment, the solid fraction was recovered by filtration and washed thoroughly with distilled water until the washes reached approximately neutral pH (pH ≈ 7), as verified with pH indicator paper. The recovered fibers were subsequently oven-dried at 60 °C for 24 h and used for the subsequent independent acid or alkaline treatment experiments.

This alkaline pretreatment constituted a common conditioning step applied to all samples before the response-surface experiments. Its purpose was to partially disrupt the lignocellulosic matrix and increase accessibility of the carbohydrate-rich fraction. Therefore, the subsequent acid and alkaline CCD treatments should be interpreted as post-pretreatment processing routes rather than as direct extraction treatments applied to native *C. palmata* fibers.

### 2.6. Chemical Treatments

The acid and alkaline treatments were evaluated as independent experimental processes according to their respective central composite designs. Each experimental run used a fresh 35 g portion of dried, pretreated *C. palmata* fiber and 100 mL of the corresponding treatment solution. The acid-treatment experiments were followed by chlorination, whereas the alkaline-treatment experiments were followed by bleaching. The operating conditions for the acid and alkaline treatments were varied according to their respective CCD matrices, while the subsequent chlorination and bleaching conditions were maintained constantly.

Following the common alkaline pretreatment, the acid and alkaline treatments were evaluated as separate post-pretreatment experimental routes using fresh portions of pretreated *C. palmata* fibers.

#### 2.6.1. Acid Treatment and Chlorination

For each experimental run, 35 g of dried pretreated fiber was treated with 100 mL of aqueous H_2_SO_4_ solution. Acid concentration (1–5%), temperature (50–120 °C), and treatment time (30–120 min) were varied according to the acid-treatment CCD (Table 3). The treatments were conducted under reflux. After acid treatment, the solid fraction was recovered by filtration, thoroughly washed with distilled water until the wash reached approximately neutral pH, and oven-dried at 60 °C for 24 h. The recovered material was then treated with 100 mL of 3.5% NaClO solution for 15 min in an ultrasonic bath (Branson 2510). Following chlorination, the solid fraction was filtered, washed with distilled water until its pH was approximately neutral, and oven-dried at 60 °C for 24 h.

#### 2.6.2. Alkaline Treatment and Bleaching

The alkaline-treatment experiments were conducted independently from the acid-treatment experiments. Each experimental run was initiated with a fresh 35 g portion of dried pretreated fiber and 100 mL of aqueous NaOH solution. NaOH concentration (10–30%), temperature (50–90 °C), and treatment time (60–180 min) were varied according to the alkaline-treatment CCD (Table 4). The treatments were conducted under reflux. After alkaline treatment, the solid fraction was recovered by filtration, thoroughly washed with distilled water until the washes reached an approximately neutral pH, and oven-dried at 60 °C for 24 h. The recovered material was then bleached with 100 mL of 0.5% NaClO solution for 90 min under continuous stirring at 1500 rpm. After bleaching, the solid fraction was filtered, washed with distilled water until its pH was approximately neutral, and oven-dried at 60 °C for 24 h before characterization.

#### 2.6.3. Gravimetric Recovery

Gravimetric recovery was calculated for each experimental run according to Equation (2):

(2) $$R\left(\%\right)=\frac{W_{f}}{W_{i}}\times 100$$

where R denotes the percentage of dry solid material retained after the complete treatment sequence relative to the initial dry mass of pretreated fiber. Accordingly, ***W_i_*** denotes the initial dry mass of pretreated fiber introduced into each experimental run (35 g), whereas ***W_f_*** denotes the dry mass of the insoluble solid fraction retained by filtration after the corresponding chemical treatment, washing, and subsequent processing steps. The term recovered material specifically refers to the solid fraction that is retained, rather than material that has been precipitated or recovered from the liquid filtrate. The biomass constituents that were solubilized or degraded during chemical treatment and then removed with the filtrate and washing liquids were not quantified separately. Therefore, gravimetric recovery indicates the retention of solid mass and should not be viewed as a measure of cellulose purity or as a direct estimate of cellulose extraction yield.

### 2.7. Characterization

Untreated *C. palmata* fibers and the materials obtained under the selected acid and alkaline treatment conditions were characterized by Fourier transform infrared spectroscopy (FTIR), X-ray diffraction (XRD), and thermogravimetric analysis (TGA) to evaluate treatment-induced changes in chemical functionality, crystalline organization, and thermal degradation behavior, respectively. The pretreated fiber was additionally analyzed by FTIR to assess changes associated with the initial alkaline pretreatment.

#### 2.7.1. Fourier Transform Infrared Spectroscopy (FTIR)

Fourier transform infrared (FTIR) spectroscopy was used to evaluate changes in the functional groups of *C. palmata* fibers associated with the chemical treatments. Prior to analysis, the samples were oven-dried at 60 °C for 24 h and finely ground to a particle size below 250 μm. The powdered samples were prepared as KBr (Sigma-Aldrich, St. Louis, MO, USA) pellets and analyzed using a Nicolet 8700 FTIR spectrometer (Thermo Fisher Scientific, Waltham, MA, USA) in transmission mode. Spectra were recorded over the wavenumber range of 4000–400 cm^−1^ at a spectral resolution of 4 cm^−1^, with 100 scans collected for each spectrum. Baseline correction and spectral normalization were performed prior to interpretation. Characteristic absorption bands were assigned to functional groups commonly associated with cellulose, hemicellulose, and lignin.

#### 2.7.2. X-Ray Diffraction (XRD)

X-ray diffraction (XRD) analysis was performed to evaluate changes in the crystalline organization of the fibers following chemical treatment. Measurements were conducted using a Bruker D8 Advance diffractometer (Bruker AXS GmbH, Karlsruhe, Germany) with Cu Kα radiation (λ = 1.5406 Å), operated at 40 kV and 30 mA. Finely ground samples (<250 μm) were scanned over a 2θ range of 5–40°, using a step size of 0.02° and a scanning rate of 1° min^−1^. The crystallinity index (CrI) was estimated using the Segal method according to Equation (3):

(3) $$CrI\left(\%\right)=\frac{I_{200}-I_{am}}{I_{200}}\times 100$$

where ***I***_200_ is the maximum intensity of the diffraction peak associated with the (200) crystalline plane at approximately 2θ = 22.5°, and ***I****_am_* is the minimum intensity assigned to the amorphous contribution at approximately 2θ = 18°.

#### 2.7.3. Thermogravimetric Analysis (TGA)

Thermogravimetric analysis (TGA) was performed to evaluate the thermal degradation behavior of untreated and chemically treated *C. palmata* fibers. Approximately 8–10 mg of dried and ground sample was placed in a platinum pan and analyzed using a TA Instruments Q500 thermogravimetric analyzer (TA Instruments, New Castle, DE, USA). Samples were heated from 25 to 600 °C at a heating rate of 10 °C min^−1^ under a nitrogen atmosphere at a flow rate of 60 mL min^−1^.

## 3. Results

### 3.1. Model Adequacy and ANOVA

The adequacy of the quadratic models fitted to gravimetric recovery data using analysis of variance (ANOVA). As summarized in Table 5, the models developed for the acid and alkaline treatments were statistically significant, with *p*-values of 0.0446 and 0.0214 and model F-values of 3.14 and 3.96, respectively. The acid-treatment model showed an R^2^ of 0.739 and an adjusted R^2^ of 0.503, whereas the alkaline-treatment model showed 0.781 and 0.584, respectively. These results indicate that the models accounted for a substantial proportion of the observed variation in gravimetric recovery; however, the differences between R^2^ and adjusted R^2^ indicate that their explanatory performance should be interpreted with caution.

The six replicated center points showed mean gravimetric recoveries of 39.57 ± 2.36% for the acid treatment and 53.77 ± 3.93% for the alkaline treatment, corresponding to coefficients of variation of 5.96% and 7.31%, respectively. This dispersion indicates non-negligible experimental variability, particularly for the alkaline route. Because center-point replication estimates pure experimental error, this variability was considered when assessing model adequacy. Accordingly, the fitted response surfaces represent overall response trends within the investigated experimental domains rather than highly precise point predictions.

For the acid treatment, H_2_SO_4_ concentration (A; *p* = 0.0260) and temperature (B; *p* = 0.0489) showed significant linear effects on gravimetric recovery, whereas treatment time (C; *p* = 0.3682) was not significant. Among the quadratic terms, only acid concentration (A^2^; *p* = 0.0084) was significant. None of the two-factor interaction terms were statistically significant. The significant quadratic contribution of acid concentration indicates curvature in the response surface, showing that the effect of H_2_SO_4_ concentration on gravimetric recovery was not adequately described by a purely linear relationship.

For the alkaline treatment, none of the linear terms for NaOH concentration, temperature, or treatment time were statistically significant (*p* > 0.05). In contrast, the quadratic terms for NaOH concentration (A^2^; *p* = 0.0054) and temperature (B^2^; *p* = 0.0041) were significant, indicating pronounced curvature of the response within the experimental domain. No statistically significant two-factor interactions were identified. These results indicate that the response to the alkaline treatment was predominantly nonlinear over the range of conditions investigated.

Numerical optimization of the fitted response surfaces identified the operating conditions of 3% H_2_SO_4_, 85 °C, and 75 min for the acid treatment and 20% NaOH, 70 °C, and 120 min for the alkaline treatment as associated with the maximum predicted gravimetric recovery within the respective experimental domains.

### 3.2. Effect of Treatment Conditions on Gravimetric Recovery

Table 6 presents the gravimetric recovery obtained under the experimental conditions evaluated in the acid and alkaline CCDs. For the acid treatment, recovery ranged from 27.5% to 42.7%, indicating that experimental conditions influenced the amount of solid material retained after chemical processing. The highest recovery was 42.7%. Consistent with the ANOVA results, the observed variation was associated primarily with changes in H_2_SO_4_ concentration and temperature, whereas treatment time did not show a statistically significant linear effect within the experimental domain.

The alkaline treatment showed a broader range of gravimetric recovery, from 19.8% to 57.7%, with the highest experimental value reaching 57.7%. This variation is consistent with the significant quadratic effects of NaOH concentration and temperature identified by ANOVA, indicating a nonlinear dependence of solid recovery on these variables. Accordingly, increasing reagent concentration or temperature did not necessarily increase gravimetric recovery within the experimental domain investigated.

Although the alkaline-treatment experiments produced a higher maximum gravimetric recovery (57.7%) than the acid-treatment experiments (42.7%), this difference should not be interpreted as direct evidence of greater cellulose purity or more extensive removal of non-cellulosic components. Gravimetric recovery reflects the amount of solid material retained after treatment and therefore depends on the combined effects of solubilization, degradation, and retention of the different constituents of the lignocellulosic matrix. Nevertheless, the distinct responses observed for the acid and alkaline treatments are consistent with the different effects of acidic and alkaline environments on lignocellulosic biomass reported in previous studies [19]. These results emphasize the importance of controlling treatment conditions to balance material recovery with the structural modifications induced by chemical processing.

A key implication of using gravimetric recovery as the response variable is that maximum solid recovery does not automatically mean maximum cellulose isolation or purity. Increasing treatment severity can enhance the solubilization or alteration of components linked to hemicellulose and lignin, but it may also reduce the amount of solid residue remaining after processing. Conversely, high gravimetric recovery may partly reflect retention of residual non-cellulosic material. Thus, this study prioritizes maintaining solid mass over achieving pure composition. The conditions identified by RSM should be understood as those linked to the highest expected gravimetric recovery within the tested experimental range, rather than the conditions that optimize cellulose purity or delignification.

The gravimetric recovery determined in the present study therefore does not directly correspond to cellulose contents or extraction yields reported for rice husk, banana residues, coconut fibers, or other lignocellulosic sources [20,21,22]. Comparisons with other lignocellulosic feedstocks should be interpreted cautiously because reported cellulose yields depend strongly on biomass composition, extraction procedure, treatment severity, and the method used to calculate yield [21,22,23,24]. Rather than establishing the superiority of *C. palmata* over these feedstocks, these results show that its response to chemical processing can be systematically modulated through the treatment conditions investigated. Together with the structural and thermal changes discussed in the following sections, these findings provide a basis for further evaluating *C. palmata* as a non-conventional lignocellulosic feedstock for cellulose-based materials.

Gravimetric recovery is a process-response variable rather than a compositional measurement. The recovered solid may contain cellulose together with residual non-cellulosic constituents, and the present experimental design did not independently quantify cellulose, hemicellulose, and lignin contents. Therefore, differences in gravimetric recovery among treatment conditions reflect the net balance between solid retention and the solubilization or degradation of lignocellulosic constituents.

### 3.3. FTIR Characterization

**Figure 2:**
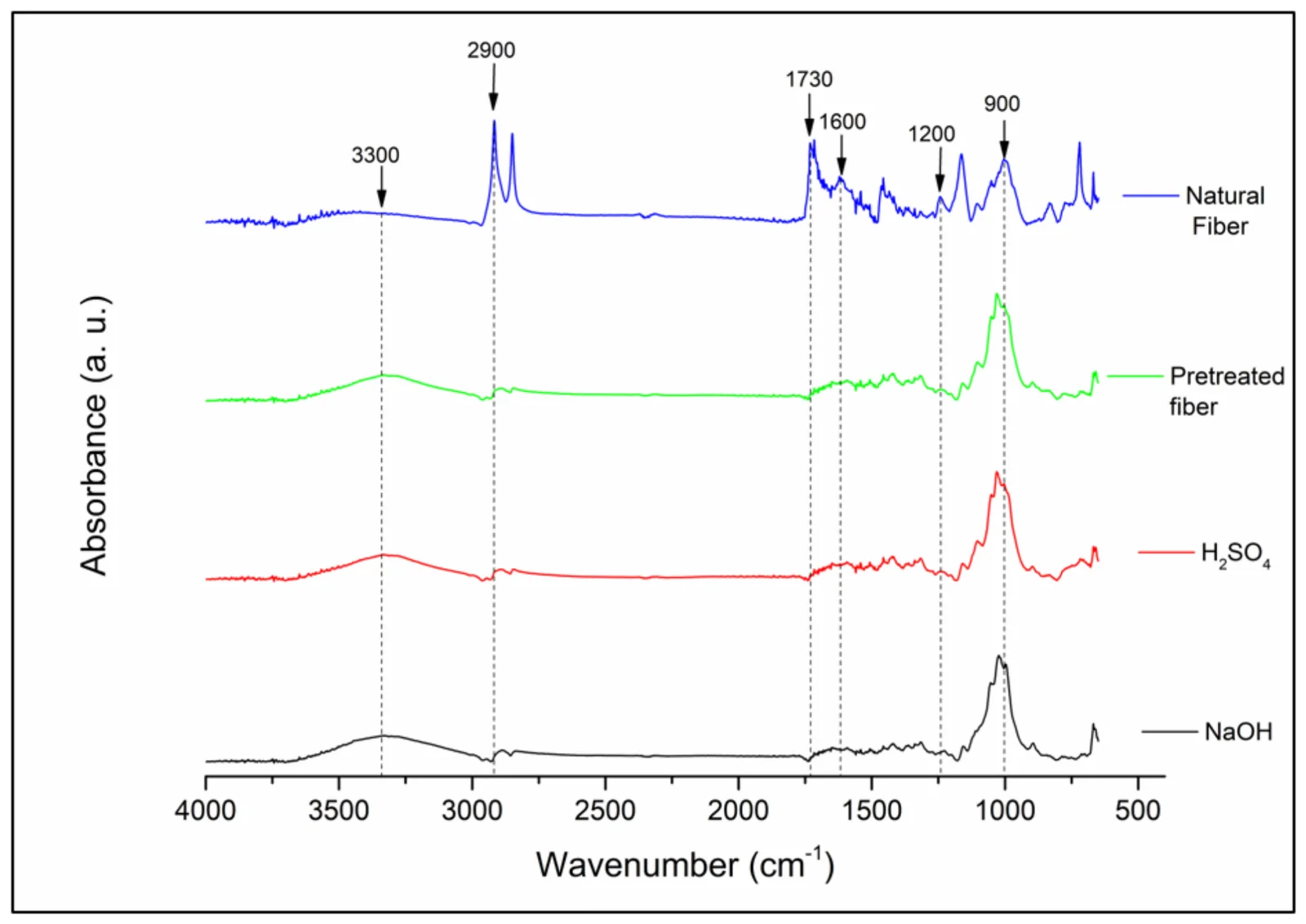
FTIR spectra of untreated, pretreated, acid-treated, and alkaline-treated *C. palmata* fibers.

The FTIR spectra of untreated, pretreated, acid-treated, and alkaline-treated *C. palmata* fibers are shown in Figure 2. The untreated fiber exhibited the characteristic absorption bands of lignocellulosic materials, including a broad band at approximately 3300 cm^−1^, attributed to O-H stretching vibrations, and a band near 2900 cm^−1^, which is associated with C-H stretching. The absorption band around 1730 cm^−1^ is commonly associated with C=O stretching of acetyl and uronic ester groups in hemicelluloses, whereas the band near 1600 cm^−1^ is associated with aromatic skeletal vibrations of lignin. The region between approximately 1200 and 900 cm^−1^ contains several overlapping vibrations associated with polysaccharide structures, including C-O-C and C-O stretching modes [25,26,27].

The pretreated fiber exhibited differences in the relative intensities of bands associated with non-cellulosic constituents compared with the untreated fiber, indicating qualitative modification of the lignocellulosic matrix during the initial alkaline pretreatment. Further spectral changes were observed after the acid and alkaline treatments. In the acid-treated material, the band near 1730 cm^−1^ showed a lower relative intensity than in the untreated fiber, together with changes in the lignin-associated region around 1600 cm^−1^. These changes are consistent with the removal or modification of hemicellulose- and lignin-associated structures during chemical treatment.

The alkaline-treated material showed lower relative intensities of bands associated with non-cellulosic components, together with the persistence of prominent absorption features in the 1000–1100 cm^−1^ region. The observed spectral changes are consistent with modification of the lignocellulosic matrix and a greater relative contribution of cellulose-associated spectral features following chemical processing. However, because FTIR provides primarily qualitative and semiquantitative information on functional groups, these spectral changes should not be interpreted as evidence of complete hemicellulose or lignin removal or as a quantitative measure of cellulose purity.

Overall, the FTIR results indicate progressive modification of the lignocellulosic matrix from the untreated fiber through pretreatment and the subsequent chemical treatments. The decrease in absorption features linked to hemicellulose and lignin, along with the continued presence of cellulose-related bands, indicates a relative increase in cellulose signals under the tested conditions. These spectroscopic observations provide complementary evidence to the gravimetric results and are further supported by the changes in crystalline organization and thermal behavior discussed in the following sections.

### 3.4. Response Surface Analysis

Response surface and contour plots were generated from the fitted quadratic models to visualize the combined effects of reagent concentration and temperature on gravimetric recovery while maintaining treatment time at the selected value for each treatment route. The resulting surfaces revealed distinct response patterns for the acid and alkaline treatments, consistent with the statistical effects identified by ANOVA. The color scale represents the predicted gravimetric recovery, with the color gradient indicating variations in the predicted response across the experimental domain.

**Figure 3:**
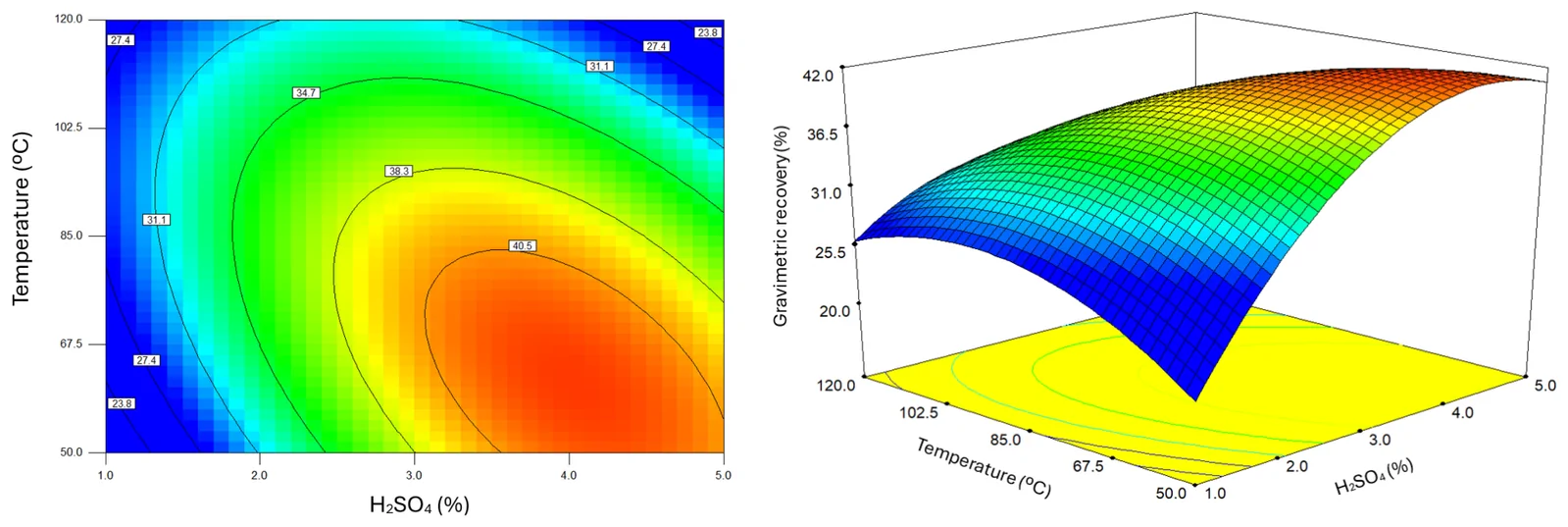
Response surface and contour plots showing the effects of acid concentration and temperature on gravimetric recovery at a fixed treatment time of 75 min.

For the acid treatment, the response surface generated at a fixed treatment time of 75 min showed a curved dependence of gravimetric recovery on H_2_SO_4_ concentration and temperature (Figure 3). Recovery increased with intermediate acid concentrations and temperatures, whereas lower values were observed with some of the more severe conditions within the experimental domain. This behavior is consistent with the significant linear effects of acid concentration and temperature and the significant quadratic effect of acid concentration identified by ANOVA. The selected operating conditions were 3% H_2_SO_4_, 85 °C, and 75 min. The decrease in gravimetric recovery observed at more severe combinations of acid concentration and temperature may reflect increased solubilization or degradation of lignocellulosic constituents; however, the gravimetric response alone does not allow this decrease to be attributed specifically to cellulose degradation.

**Figure 4:**
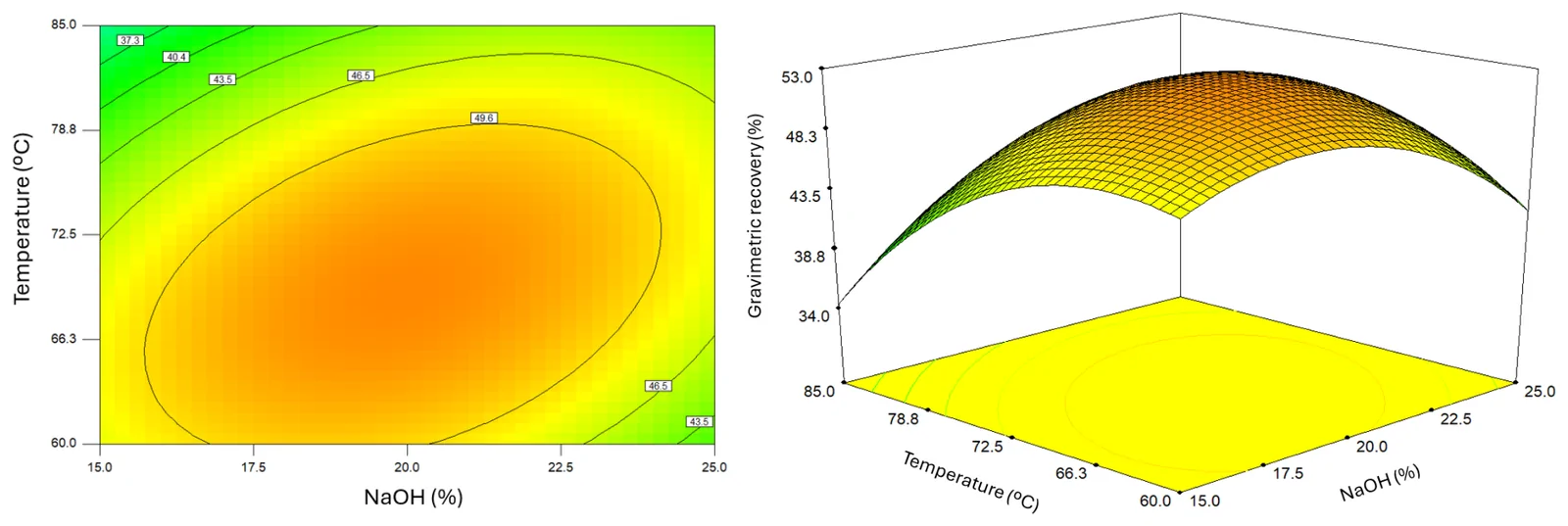
Response surface and contour plots showing the effects of alkaline concentration and temperature on gravimetric recovery at a fixed treatment time of 120 min.

For the alkaline treatment, the response surface generated at a fixed treatment time of 120 min also exhibited pronounced curvature (Figure 4). Higher gravimetric recovery was observed within an intermediate region of NaOH concentration and temperature, whereas the response decreased toward the boundaries of the experimental domain. This behavior agrees with the significant quadratic effects of NaOH concentration and temperature identified by ANOVA. The selected operating conditions were 20% NaOH, 70 °C, and 120 min, corresponding to the region associated with maximum predicted gravimetric recovery within the investigated domain.

Overall, the response surfaces show that nonlinear effects of the treatment variables governed gravimetric recovery and that increasing chemical concentration or temperature did not necessarily increase recovered material. Importantly, the acid and alkaline response surfaces represent independent experimental routes and should not be interpreted as consecutive stages of a single extraction sequence. The RSM analysis therefore identified operating regions associated with favorable gravimetric recovery for each treatment route within the experimental ranges investigated.

Therefore, in this study, optimization specifically pertains to gravimetric recovery. Because cellulose, hemicellulose, and lignin contents are not listed as quantitative response variables, the response surfaces cannot determine the optimal conditions for obtaining a high cellulose purity or selective delignification. A multi-response optimization that considers both material recovery and quantitative composition is necessary to determine the conditions that concurrently maximize cellulose retention while removing non-cellulosic components.

To assess agreement between the model predictions and the experimental response, we conducted independent confirmation experiments under the operating conditions identified by numerical optimization. For the acid treatment, the model predicted a response of 42.06%, whereas the experimental confirmation runs (*n* = 3) yielded a mean response of 44.43%. For the alkaline treatment, the model predicted 55.42%, while the experimental confirmation runs (*n* = 3) yielded a mean response of 58.20%. These values correspond to prediction errors of 5.63% and 5.02% for the acid and alkaline treatments, respectively, equivalent to prediction-experiment agreements of 94.37% and 94.98%. The small deviations between predicted and experimentally observed responses support the fitted models’ ability to approximate the experimental response under the selected conditions. Table 7 summarizes the validation results.

### 3.5. X-Ray Diffraction (XRD) Analysis

**Figure 5:**
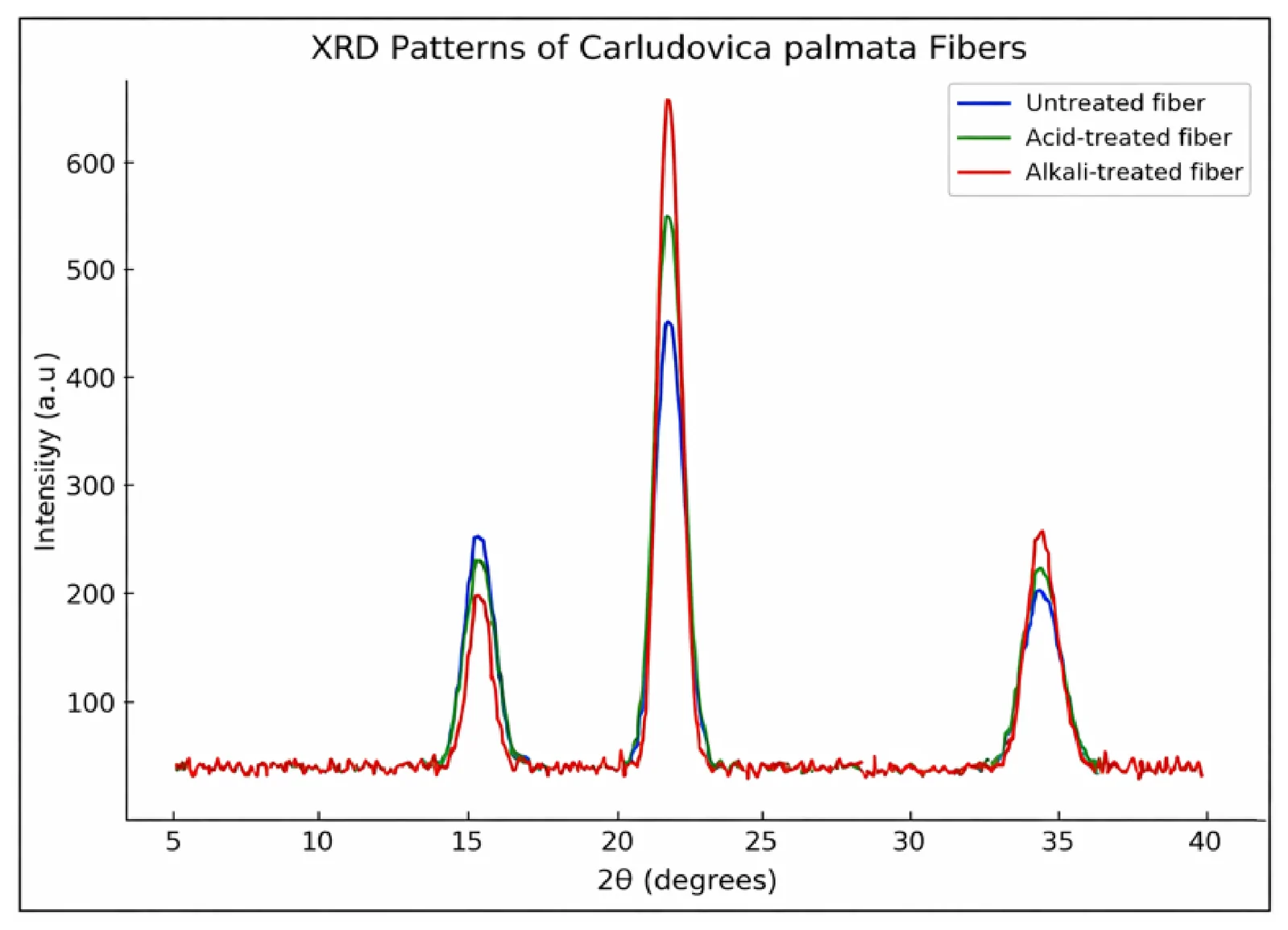
X-ray diffraction patterns of untreated *C. palmata* fibers and materials obtained under the selected acid and alkaline treatment conditions.

The XRD patterns of untreated *C. palmata* fibers and the materials obtained under the selected acid and alkaline treatment conditions are shown in Figure 5. All samples showed diffraction features characteristic of cellulose I, with reflections at approximately 2θ = 15.8°, 22.4°, and 34.6°, corresponding to the (110)/(110), (200), and (004) crystallographic planes, respectively. The persistence of these reflections after chemical treatment indicates that the characteristic cellulose I crystalline structure was retained under the investigated processing conditions.

The crystallinity index (CrI), estimated using the Segal method, increased from 41.2% for the untreated fiber to 52.7% for the material obtained under the selected acid-treatment conditions and 63.8% for the material obtained under the selected alkaline-treatment conditions. These values correspond to increases of 11.5 and 22.6 percentage points, respectively, relative to the untreated fiber. The increase in CrI after chemical treatment is consistent with a reduction in the relative contribution of amorphous constituents within the lignocellulosic matrix, including hemicellulose and lignin, thereby increasing the relative contribution of the crystalline cellulose fraction to the diffraction pattern [26].

The higher CrI observed for the alkaline-treated material compared with the acid-treated material suggests a greater relative contribution of ordered cellulose domains after alkaline processing under the conditions investigated. This interpretation is consistent with the FTIR results, which showed reductions in absorption features associated with non-cellulosic constituents. However, because the Segal method provides a semiquantitative estimate based on diffraction intensities, the calculated CrI values should not be interpreted as absolute measurements of cellulose crystallinity or cellulose purity.

Overall, the XRD results indicate that both chemical treatment routes modified the structural organization of the *C. palmata* fibers while preserving the characteristic cellulose I diffraction pattern. The increase in the estimated crystallinity index, together with the FTIR spectral changes, supports the relative enrichment of cellulose-associated structural features in the chemically treated materials.

### 3.6. Effect of Chemical Treatment on Thermal Degradation Behavior

**Figure 6:**
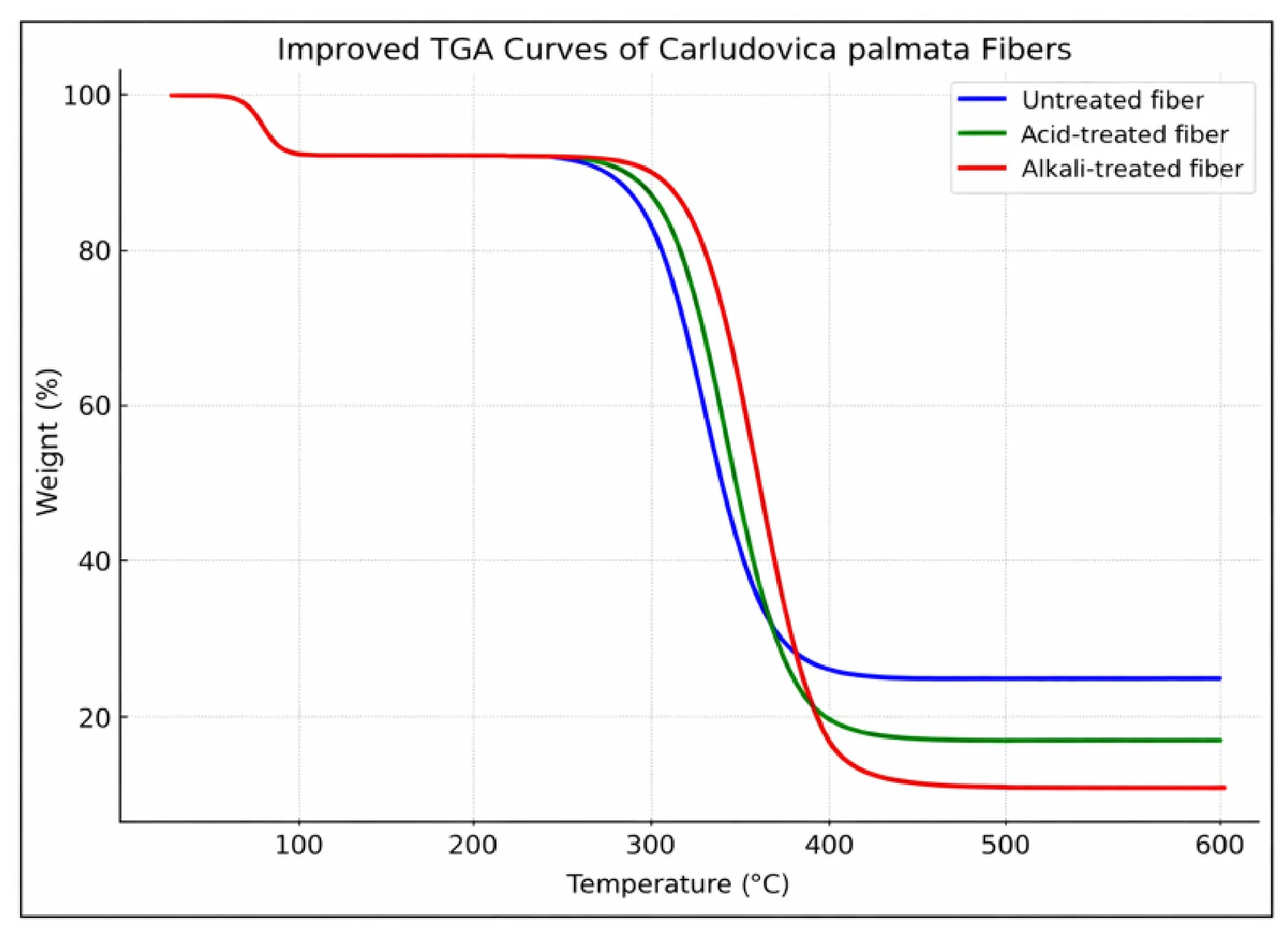
Thermogravimetric curves of untreated *C. palmata* fibers and materials obtained under the selected acid and alkaline treatment conditions.

Figure 6 shows the thermal degradation behavior of untreated *C. palmata* fibers and materials obtained under the selected acid and alkaline treatment conditions. The untreated fiber showed an initial mass loss below approximately 120 °C, primarily due to the removal of absorbed moisture and low-molecular-weight volatile components [28,29]. The main degradation stage occurred between approximately 220 and 360 °C, with a maximum degradation temperature T_max_ of approximately 328 °C.

The treated samples exhibited the main degradation event at higher observed temperatures than the untreated fiber under the analyzed conditions. Under the selected acid-treatment conditions, the material showed a T_max_ of approximately 335–340 °C, whereas among the analyzed samples, the alkaline-treated material showed the highest observed T_max_, approximately 345–350 °C. These changes suggest increased thermal stability under the tested conditions. The observed changes are consistent with modification of the lignocellulosic matrix and the relative enrichment of the cellulose-associated fraction following chemical processing.

Differences were also observed in the residual mass remaining at the end of the thermal analysis. The acid-treated material showed a residual mass of approximately 21%, whereas the alkaline-treated material exhibited a residual mass of approximately 15–18%. Residual mass after thermal degradation can be influenced by several factors, including char formation, inorganic constituents, and differences in the composition and degradation pathways of the treated materials [30]. Therefore, these values should not be interpreted as direct quantitative measures of cellulose, lignin, or other individual constituents.

Overall, the TGA results indicate that the chemical treatments modified the thermal degradation behavior of *C. palmata* fibers, with both treated materials exhibiting higher maximum degradation temperatures than the untreated fiber. The alkali-treated sample showed the most significant change in degradation temperature among all samples analyzed. These results, combined with FTIR and XRD data, support the idea that the treatment causes changes in the structural and thermal properties of the recovered materials.

## 4. Conclusions

Response surface methodology based on central composite designs was applied to evaluate the effects of reagent concentration, temperature, and treatment time on the gravimetric recovery of *Carludovica palmata* fibers subjected to independent acid and alkaline treatment routes. The fitted models identified distinct nonlinear responses to the processing variables and enabled the selection of operating conditions associated with maximum predicted gravimetric recovery within the respective experimental domains. Maximum experimental recoveries of 42.7% and 57.7% were obtained for the acid and alkaline treatments, respectively.

Chemical treatment also produced measurable changes in the structural and thermal characteristics of the recovered materials. FTIR analysis showed reductions in absorption features associated with hemicellulose- and lignin-related functionalities, while XRD analysis showed an increase in the Segal crystallinity index from 41.2% in the untreated fiber to 52.7% and 63.8% in the acid- and alkaline-treated materials, respectively. TGA also showed a shift in the maximum degradation temperature toward higher values after chemical treatment, with the alkaline-treated material showing the greatest increase under the conditions investigated. Collectively, these results indicate that the treatment conditions affect not only the amount of recovered material but also its chemical functionality, crystalline organization, and thermal degradation behavior.

The gravimetric recovery values reported in this study should not be interpreted as direct measurements of cellulose content or purity because we did not perform quantitative compositional analysis of cellulose, hemicellulose, and lignin. Similarly, the spectroscopic and diffraction results provide complementary evidence of cellulose enrichment rather than a quantitative determination of chemical composition. Further studies incorporating quantitative compositional analysis, morphological characterization, and degree-of-polymerization measurements would provide a more complete assessment of the recovered cellulose-rich materials. Their incorporation into films, composites, or other polymer systems should also be investigated to establish structure-property relationships and determine their suitability for specific cellulose-based applications.

Therefore, the conditions found through RSM indicate optimal points for gravimetric recovery, not for achieving higher cellulose purity. Future research incorporating standardized quantitative compositional analysis as an extra response could facilitate multi-objective optimization, balancing solid recovery with the selective removal of non-cellulosic components.

## Figures and Tables

**Table 1:** Coded and actual levels of the independent variables used in the CCD for the acid treatment.

Independent Variable	Coded Values				
	−1.68179	−1	0	1	1.68179
A	1	2	3	4	5
B	50	67.5	85	102.5	120
C	30	52.5	75	97.5	120

**Table 2:** Coded and actual levels of the independent variables used in the CCD for the alkaline treatment.

Independent Variable	Coded Values				
	−1.68179	−1	0	1	1.68179
A	10	15	20	25	30
B	50	60	70	80	90
C	60	90	120	150	180

**Table 3:** Central composite design matrix for the acid treatment.

Design-Expert Code				Real Treatment (H_2_SO_4_)		
Run	A (%)	B (°C)	C (min)	A (%)	B (°C)	C (min)
1	−1.68179	0.00	0.00	1	85	75
2	1.00	−1.00	1.00	4	67.5	97.5
3	1.00	1.00	1.00	4	102.5	97.5
4	0.00	0.00	0.00	3	85	75
5	−1.00	1.00	−1.00	2	102.5	52.5
6	0.00	−1.68179	0.00	3	50	75
7	0.00	0.00	1.68179	3	85	120
8	1.00	−1.00	−1.00	4	67.5	52.5
9	1.00	1.00	−1.00	4	102.5	52.5
10	0.00	1.68179	0.00	3	120	75
11	0.00	0.00	0.00	3	85	75
12	0.00	0.00	0.00	3	85	75
13	1.68179	0.00	0.00	5	85	75
14	−1.00	1.00	1.00	2	102.5	97.5
15	0.00	0.00	0.00	3	85	75
16	0.00	0.00	0.00	3	85	75
17	0.00	0.00	0.00	3	85	75
18	0.00	0.00	−1.68179	3	85	30
19	−1.00	−1.00	1.00	2	67.5	97.5
20	−1.00	−1.00	−1.00	2	67.5	52.5

**Table 4:** Central composite design matrix for the alkaline treatment.

Design-Expert Code				Real Treatment (NaOH)		
Run	A (%)	B (°C)	C (min)	A (%)	B (°C)	C (min)
1	0.00	0.00	−1.68179	20	70.0	60
2	1.68179	0.00	0.00	30	70.0	120
3	1.00	−1.00	−1.00	25	60.0	90
4	0.00	1.68179	0.00	20	90.0	120
5	−1.00	1.00	1.00	15	80.0	150
6	1.00	1.00	1.00	25	80.0	150
7	−1.00	1.00	−1.00	15	80.0	90
8	0.00	0.00	0.00	20	70.0	120
9	0.00	0.00	0.00	20	70.0	120
10	0.00	0.00	0.00	20	70.0	120
11	1.00	−1.00	1.00	25	60.0	150
12	0.00	0.00	1.68179	20	70.0	180
13	−1.00	−1.00	1.00	15	60.0	150
14	−1.68179	0.00	0.00	10	70.0	120
15	0.00	0.00	0.00	20	70.0	120
16	−1.00	−1.00	−1.00	15	60.0	90
17	1.00	1.00	−1.00	25	80.0	90
18	0.00	0.00	0.00	20	70.0	120
19	0.00	−1.68179	0.00	20	50.0	120
20	0.00	0.00	0.00	20	70.0	120

**Table 5:** Statistical parameters of quadratic response surface models for assessing gravimetric recovery of acid- and alkaline-treated *Carludovica palmata* fibers.

Parameter	Acid Treatment	Alkaline Treatment
Model F-value	2.28	4.93
Model *p*-value	*p* = 0.0446 (significant)	*p* = 0.0214 (significant)
R^2^	0.739	0.781
Adjusted R^2^	0.503	0.584
PRESS	724.14	2771.06
Lack-of-fit F-value	3.03	4.53
Lack-of-fit *p*-value	0.1248	0.0615
Pure error df	5	5
Lack-of-fit df	5	5
Approx. Adequate Precision	5.19	6.77
Center-point mean ± SD (%)	39.60 ± 2.39	53.77 ± 3.93
Center-point CV (%)	6.02	7.31
Maximum Cook’s distance	0.996	0.834
Run with maximum Cook’s distance	5	11
Observations with Cook’s D > 1	None	None
Shapiro–Wilk *p*-value for studentized residuals	0.704	0.784
Residual normality	No significant departure detected	No significant departure detected

**Table 6:** Gravimetric recovery obtained for the acid and alkaline treatment experiments.

Run	Gravimetric Recovery, R (%)	
	Acid Treatment	Alkaline Treatment
1	27.5	51.8
2	38.3	39.5
3	39.2	43.5
4	37.3	19.8
5	35.8	30.5
6	41.0	35.3
7	39.5	37.2
8	42.5	57.7
9	34.8	55.5
10	27.8	57.6
11	36.5	24.8
12	42.7	52.5
13	35.2	37.0
14	36.5	31.8
15	39.3	48.3
16	40.4	51.3
17	41.2	45.8
18	36.5	53.5
19	35.5	47.3
20	31.0	50.0

**Table 7:** Experimental validation of the operating conditions predicted by the model for both acid and alkaline treatments.

Treatment	Predicted Value (%)	Experimental Value (%)	n	Error (%)	Concordance (%)
Acid	42.06	44.43	3	5.63	94.37
Alkaline	55.42	58.20	3	5.02	94.98

Experimental values correspond to the mean of three independent confirmation runs (*n* = 3).

## Data Availability

All data generated or analyzed during this study are included in this published article.
